# Platelets as a potential new immune coordinator in T cell-mediated aplastic anemia

**DOI:** 10.3389/fonc.2025.1568169

**Published:** 2025-06-09

**Authors:** Shuai Tan, Huizhen He, Yuxin Li, Mingyue Shang, Yaofang Cao, Dongmei Zou, Ronghua Hu, Wuhan Hui, Xiaoli Chang, Jing Ni, Qiang Ma, Li Su, Jing Sun, Wanxue He, Xingmin Feng, Wanling Sun

**Affiliations:** ^1^ Department of Hematology, Xuanwu Hospital, Capital Medical University, Beijing, China; ^2^ Department of Pulmonary and Critical Care Medicine, Xuanwu Hospital, Capital Medical University, Beijing, China; ^3^ National Heart, Lung, and Blood Institute, National Institutes of Health, Bethesda, MD, United States

**Keywords:** aplastic anemia (AA), CD8 + T cell/CTL, CD4 + T cell, platelet, mitochondria, immunology

## Abstract

Acquired aplastic anemia (AA) is a bone marrow failure syndrome characterized by pancytopenia and decreased hematopoietic stem and progenitor cells (HSPCs) in the bone marrow, it can be either congenital or acquired, predominantly affecting adolescents and the elderly, with higher incidence in Asia compared to Europe and America. Current treatment options include allogeneic hematopoietic stem cell transplantation or immunosuppressive agents, yet proximately a third of patients fail to reach long-term survival. AA is primarily driven by immune-mediated destruction of HSPCs, initiated by self-activated T cells. Early stages feature a Th1 response, which later shifts to Th17 and effector memory CD8^+^ T cells. Key cytokines including interferon-gamma (IFN-γ) and tumor necrosis factor-alpha (TNF-α) play crucial roles in this immune dysregulation, influencing HSPCs and contributing to bone marrow failure. Furthermore, bone marrow macrophages (MΦ), particularly M1 subtype, are implicated in AA via the TNF-α/TNF-α receptor pathway, leading to T cell activating and subsequent HSPC damage. Interestingly, MΦ with high expression of IL-27Ra have been demonstrated to contribute to HSPC destruction in AA murine models. Beyond their role in thrombosis, platelets also participate in immune regulation. Some studies suggest that platelet may modulate T cell responses through mechanisms such as Akt-PGC1α-TFAM pathway or PF4-mediated activity, which could play a role in AA. However, direct evidence connecting platelet regulation to T cell-mediated HSPC damage is limited, and current research has largely focuses on CD8^+^ T cells. Moving forward, it is essential to investigate the interactions between platelets, CD4^+^ T cells, and mitochondrial energy metabolism. In this review, we propose that platelet-derived factors such as PF4 and TGFβ may activate mitochondrial pathways, influencing T cell activation and leading to HSPC destruction in AA. This hypothesis could provide new insights into the molecular mechanisms of AA and pave the way for novel therapeutic strategies (Highlight).

## Highlights

- This review will discuss and explore the pathogenesis of AA from a new perspective, focusing on platelet-regulated T-cell immune response.
- The function of platelets are not only thrombus and hemostasis, but also regulate T-cell immunity. The key mediators involved are PF4 and the mitochondrial energy metabolism signaling pathway.
- We also summarize the research history of platelet drugs in cardiovascular and cerebrovascular diseases, as well as the potential of immunotherapy in the current era.
- Molecular Activity of Platelet-Regulated T Cell Immune Response in AA as below:

## Introduction

Aplastic Anemia (AA) may arise from an unknown pathogen infecting hematopoietic stem cells (HSCs) or peripheral cells, leading to the presentation of pathogen particles and either unmodified or chemically/genetically modified components on their cell surfaces. These antigens are subsequently processed by antigen-presenting cells (APCs) and presented to CD4^+^ T cells. Platelets play a role in this immune regulation by releasing various soluble mediators, such as PF4/CXCL4, TGFβ, PAF, TXA2, NAP, and RANTES. PF4 binds to CXCR3 and CXCR5 receptors on CD4^+^ or CD8^+^ T cells, activating downstream mitochondrial energy metabolism signaling pathway (Akt-PGC1α-TFAM). This affects mitochondrial quantity, ATP production, and reactive oxygen species (ROS), ultimately regulating T cell immune response. In CD4^+^ T cells, this regulation leads to: ①Differentiation into Th1, Th2, Th9, and Th22 phenotypes; ②Simultaneous differentiation into Th17 phenotypes under the stimulation of IL-23 and IL-12; ③Suppression of Treg cells, resulting in weakened immune regulation and imbalance. In CD8^+^ T cells, the immune regulation involves: ①Direct cytotoxicity through the release of granzyme B (GzmB) and perforin (PFN); ②Paracrine effects via secretion of TNFα, IFNγ, and Fas ligand (Fas-L).

In summary, platelets regulate the immune responses of CD4^+^ and CD8^+^ T cells through mitochondrial energy metabolism, contributing to immune dysregulation and increased levels of IFNγ and TNFα, leading to the destruction of HSPCs and potential bone marrow failure.

While platelets are best known for their role in hemostasis and thrombosis, recent evidence suggests that platelets release mediators such as PF4/CXCL4 (platelet factor 4), TGFβ (transforming growth factor β), RANTES, and others through α-particle secretion. These processes, mediated by pathways like mitochondrial Akt-PGC1α and Toll-like receptors (TLR), selectively regulate T-cell recruitment under normal blood flow, affecting the lifespan of naive naïve T cells (TN) and memory T cells (TM). Moreover, platelets influence the dynamics and differentiation of Th (T helper) and Treg (regulatory T) cells, thus playing a critical role in immune regulation (1–6).

AA is characterized by bone marrow failure, pancytopenia, and reduced bone marrow cellularity. It most commonly affects in adolescents and the elderly, with a higher incidence in Asia than in Europe and the United States. Immunosuppressive therapy is the primary treatment, but it has a slow onset and significant side effects, with about a third of patients not surviving long term. Recent advances have seen TPO-RAs (thrombopoietin receptor agonists) combined with IST as a first-line treatment, though whether TPO-RAs can correct immune imbalances while promoting platelet production remains unclear (7–9).

Research shows that patients with AA exhibit abnormal T-cell activation, characterized by elevated levels of IFN-γ and TNF-α, leading to the destruction of HSPCs. Identifying the factors driving excessive T-cell activation and developing effective strategies to correct immune imbalance remain crucial challenges (10–13).

This paper proposes a novel clinical perspective: investigating the role of platelet-mediated T-cell immune function in bone marrow failure, potentially offering innovative approaches to addressing treatment challenges in the future.

## Platelet physiology

Platelets are anucleate, discoid cells with dimensions of approximatley (2.0-5.0)×0.5 µm, an average cell volume of 6–10 fl, a blood concentration of 200-300×10^9^/L, and a lifespan of approximately 10 days (14). Though primarily known for their role in hemostasis and thrombosis, platelets are critical in conditions such as myocardial infarction (MI), which is frequently caused by thrombus formation. They are also implicated in immune system disorders, including viral and bacterial infections in patients with idiopathic thrombocytopenic purpura (15). Emerging evidence indicates that platelets also contribute to immune functions through Toll-like receptors (TLR) (1, 16–19).

Upon activation, platelets release α-granules, which are linked to major histocompatibility complex I (MHCI) molecules. MHCI expression can be found on the platelet plasma membrane and in the cytosol, although it is relatively unstable in these areas (20, 21). In addition to MHCI, α-granules release mediators like PF4/CXCL4 and TGFβ, that play essential roles in thrombosis, inflammation, and immune modulation, while also contributing to vascular intimal injury (22). PF4 exhibits a strong chemotactic effect on neutrophils by binding to heparan sulfate on vascular endothelium, promoting thrombin activation. Additionally, circulating TGFβ primarily originate from platelets, serving as a specific marker of *in vivo* platelet activation (2, 23–25).

Atherosclerosis, a chronic inflammatory process, involves an sustained immune response. Platelets are involved in all stages of thrombus formation, which encompasses four key stages: endothelial dysfunction, fatty streak formation, advanced and complicated lesions, and emergence of unstable fibrous plaques (26). For the prevention and treatment of atherosclerotic diseases, antiplatelet therapy has long been a cornerstone, with a consensus on its benefits (27). Current therapies mainly target direct platelet activation, using agents like COX-inhibitor aspirin and ADP P2Y12 receptor antagonists such as clopidogrel or ticagrelor. These treatments reduce acute coronary syndrome risks by approximately 30-40%. However, efforts to further enhance antiplatelet therapy have reached a therapeutic plateau, as illustrated by ticagrelor’s marginal 2% superior over clopidogrel in primary endpoint protection over one year (28, 29).

This highlights the need for novel antiplatelet strategies. Future therapies may focus on platelet interactions with inflammatory and immune processes. A combination of antiplatelet drugs that target both platelet activation and inflammation could synergistic effects, offering more comprehensive protection against thrombotic and immune-related complications.

Platelets exhibit immunomodulatory potential, as evidenced by their ability to stimulate lymphocyte proliferation, optimize the subpopulation ratio of lymphocytes by regulating the CD3+CD8+ T lymphocytes and bolster the cytotoxic function of lymphocytes *in vitro* when activated (30). Additionally, platelets inhibit cytolytic function, adhesion ability and cytotoxic properties of NK cells by expressing glucocorticoid-induced TNF-related ligand (GITRL), releasing TGF-β and transferring MHC-I, thereby protecting tumor cells and promoting the formation of immunosuppressive environment (6, 31, 32). Moreover, platelets play a role in modulating the balance of macrophage phenotypes. Studies have shown that platelets can promote the polarization of macrophages to M1 phenotype in a mouse model of septic shock *in vitro* (33, 34). Meanwhile, related research has also indicated that thrombopoietin receptor agonists induce macrophages to shift towards the M2 phenotype (35). These discoveries not only enhance our understanding of the immune-regulating role of platelets, but also offer fresh perspectives and approaches for the treatment of associated diseases.

One of the characteristics of aplastic anemia is decrease in platelets. Thrombopoietin receptor agonists (TPO-RAs) regulate the differentiation and maturation of megakaryocytes required for platelet production. So thrombopoietin receptor agonists have been used to treat bone marrow failure syndromes, such as aplastic anemia (36). Eltrombopag is one of TPO-RAs that improves the blood platelet level. A two-arm study suggests patients with severe aplastic anemia (SAA) and very severe aplastic anemia (vSAA) who do not have a suitable bone marrow transplant donor need standard immunosuppression treatment. Compared with Group A (the standard immunosuppressive therapy), more participants in group B(the standard immunosuppressive therapy with Eltrombopag) have elevated blood cell levels into the normal range and responded more quickly to treatment, while side effects were similar in both groups. So immunosuppression treatment with eltrombopag benefits participants with SAA and vSAA (37). And the recovery of platelet level plays an important role in the treatment of aplastic anemia.

## CD4^+^ and CD8^+^ T cells

### CD4^+^ T cells

Antigen-presenting cells (APCs) form a major histocompatibility complex II (MHCII)-peptide complex on their surface. This complex can be recognized and bound by the T cell receptor (TCR) and CD4, which constitutes the first signal for CD4^+^ T cell activation. The second signal involves costimulation from either cytokines or membrane proteins such as B7 (CD80) on the APC surface interacting with CD28 on the CD4^+^ T cell surface. Without this costimulation, CD4^+^ T cells remain energy-deprived. When CD4^+^ T cells are activated, they start to synthesize and secrete IL-2, leading to their proliferation and differentiation. Most activated CD4^+^ T cells become effector cells and eventually die, but a few will survive to become memory cells, retaining the long-term memory of the antigen. The first and second signals are critical for T cell survival and proliferation, but they’re insufficient for complete functional differentiation. The third signal, which drives differentiation, comes from cytokines secreted by other cells in the surrounding microenvironment, which, along with TCR recognition, determines the final outcome of CD4^+^ T cells (38, 39).

### CD4^+^ Th and Treg cells

CD4^+^ T cells can differentiate into various Th cell subsets, such as Th1, Th2, Th9, Th17, Th22, TFH, TFR, and Treg cells. These subsets play distinct roles in the immune system function of eliminating pathogens and are influenced by specific transcription factors like T-bet, GATA3, RORγt, and FoxP3 (40, 41). Additionally, certain chromatin modifiers can affect T cell function at the gene level (42).

### Th1 and Th2

Under normal physiological conditions, Th1 and Th2 cells maintain a balanced state (43, 44). Th1 cells primarily secrete pro-inflammatory cytokines like IFNγ, which mediate cellular immunity and activate other cell types, such as macrophages (45–49). Th2 cells, on the other hand, support the survival and function of B cells, including their proliferation, maturation, antibody production, and mediation of humoral immunity. This is achieved through the secretion of IL-4, IL-5, and IL-13 by Th2 cells. It’s worth noting that these two subsets regulate each other to maintain immune homeostasis; IL-4 and IFNγ can inhibit the functions of Th1 and Th2 cells, respectively (50). There is also a specialized subtype of “Th2/Th1” cells that express both IL-4 and IFNγ, along with GATA3 and T-bet. Intriguingly, antigens like Papain and House Dust Mites (HDM) can induce Th2 cells through non-MHC-II pathways, suggesting an additional role for Th2 cells in adaptive immunity (51, 52).

### Th9

Under the influence of IL-2-induced STAT5 activation, along with the regulation by IL-4 and TGF-β, CD4+ T cells can polarize toward the Th9 phenotype (53, 54). Conversely, GFI1 acts as a negative regulator for Th9 polarization. Key downstream transcription factors like PU.1, IRF4 (Interferon Regulatory Factor 4), Id1, and HIF1α are crucial for Th9 differentiation (55–57). The hallmark cytokine produced by Th9 cells is IL-9, which is closely associated with allergies and autoimmunity. TGF-β promotes IL-9 production by activating PU.1 or by facilitating the interaction between Smad2/3 and IRF4 through the TGF-β/Smad axis. Id1, when bound with Tcf3/4 or IRF4, enhances IL-9 expression in Th9 cells by interacting with the IL-9 promoter region (58). Animal and *in vitro* experiments have shown that DBP can increase IL-9 gene expression, while EB28 does the opposite (59). When IL-9 binds to its receptor, it can activate three distinct STAT proteins: STAT1, STAT3, and STAT5, which play unique roles in gene induction, differentiation, and inhibition of apoptosis (60–65).

### Th17

Th17 cells are characterized by their expression of IL-17 (IL-17^+^ IFNγ^–^) (66, 67). IL-17 is a key pro-inflammatory factor with multiple functions, such as stimulating neutrophil proliferation and maturation and inducing pro-inflammatory cytokine expression in various cell types. Because of this, Th17 cells can affect the pathophysiology of several conditions, including infections, cancer, autoimmunity, aplastic anemia, rheumatoid arthritis (RA), and others (68–71).

### Th22

Th22 cells are marked by their high production of IL-22, which is regulated by the NOTCH-HES-1 axis and the characteristic expression of chemokine receptors CCR4, CCR6, and CCR10 (72–74). IL-22 activates several downstream pathways, including MAPK, PI3K/Akt, and NF-κB, enabling it to perform various functions (75–78). While Th22 cells were initially found in studies of skin pathophysiology, recent research has shown that they are involved in various autoimmune diseases, viral infections, cardiovascular diseases, and tumors.

### TFH (CXCR5, CXCR13/BCA-1, ICOS, IcosL, Bcl6, CD40L; CD40, IL-21, PD-1)

TFH (T follicular helper) cells are characterized by the expression of CXCR5 (CXC chemokine receptor 5). Various factors play roles in the multi-stage regulation of TFH differentiation (79). CXCR5, ICOS (Inducible costimulator), IL-12, IL-21, IFNγ, IL-27, and IL-6 positively regulate TFH differentiation, while PD-1 (Programmed Death 1), CTLA-4 (Cytotoxic T Lymphocyte Antigen 4), ubiquitin ligase Peli1, IL-2, and IL-7 have the opposite effects (80–86). TFH cells primarily assist in the formation of germinal centers (GCs) and B cell differentiation and maturation, closely tied to the humoral immune response (87–89).

### TFR (CXCR5, ICOS, Bcl6, PD-1; CTLA4, GITR, FOXP3)

In 2011, a specialized subset of regulatory T cells (Tregs), located in the germinal center (GC), was identified as Tfr cells (90). Similar to Tregs, this subset contains key molecules such as FoxP3, CTLA-4, GITR, Prdm1, and Blimp-1. Additionally, Tfr cells express Bcl-6, CXCR5, PD-1, and ICOS, similar to TFH cells, distinguishing them as a unique CD4 T-cell subset (91–98). The primary function of Tfr cells is to balance immune activation and tolerance, which provides insight into autoimmune diseases, allergic reactions, antibody-mediated rejection, viral infections, and type 1 diabetes (99–102).

### Treg cells

Treg cells constitute an immunosuppressive T cell subset the production of which is induced by IL-2-stimulated FoxP3 transcriptional activity. This subset plays a crucial role in maintaining peripheral immune tolerance and controlling autoimmune responses (103–115). The balance between Treg and other Th subsets is crucial in preventing autoimmunity by impeding the activation of autoreactive T cells and the expression of cytokines (116, 117). Tregs express IL-10 and TGFβ, which inhibit macrophage function after TLR4 activation (118–120). IL-10 downregulates T cell-mediated immune responses, including repressing the proliferation of Th1 and Th2 cells, while TGFβ regulates the functions of various immune cells (121–124).

**Figure 1:**
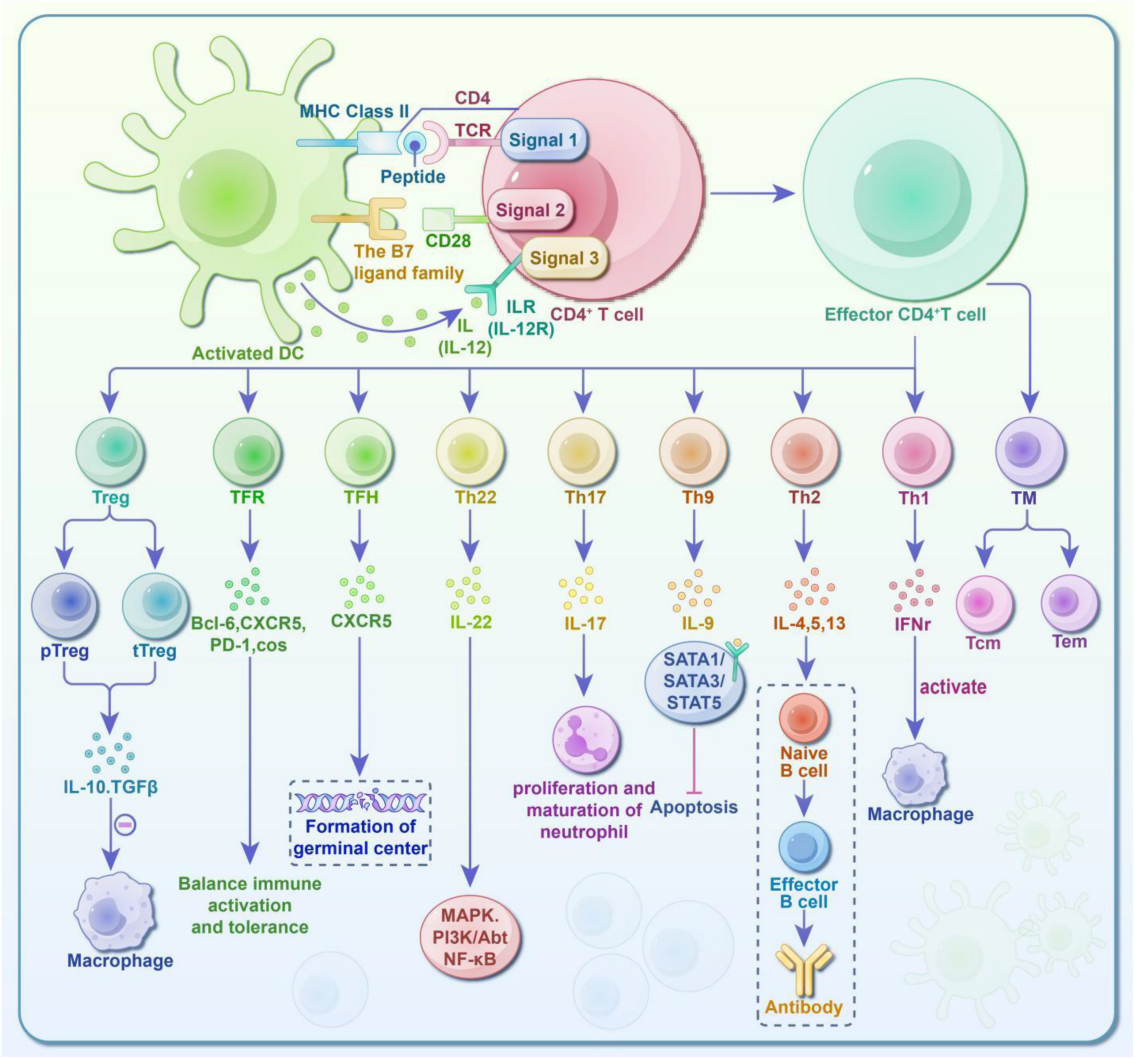
Diagram of CD4^+^ T cell subsets and immune response activity. CD4^+^ T cells require three sequential signals to activate and acquire the ability to differentiate and function: ① First Signal: Antigen-specific interactions. The CD4 co-receptor and TCR-CD3 complex recognize the antigen-MHC II complex on antigen-presenting cells (APCs), enabling signal transmission. ② Second Signal: Co-stimulatory molecules. The TCR binds to the MHC II complex, while co-stimulatory molecules on the APC surface bind to ligands on the T cell surface. ③ Third Signal: Instructive cytokines. While the first and second signals activate the T cells (promoting survival and proliferation), they are insufficient for functional differentiation. The third signal, provided by cytokines secreted by surrounding cells, together with the TCR, determines the final differentiation of the CD4^+^ T cells. Fully activated T cells can differentiate into various subsets: T Helper Cells (Th): These promote immune responses and include Th1, Th2, Th9, Th17, Th22, TFH, and TFR cells. Regulatory T Cells (Treg): These inhibit and regulate immune responses. Their specific roles are depicted in the diagram. Additionally, some effector T cells transition into memory T cells (TM), which are further classified into central memory T cells (Tcm) and effector memory cells (Tem). Upon encountering pathogens again, memory T cells produce rapid and robust immune responses, which are crucial for long-term immunity and vaccine effectiveness.

In summary, the diagram of CD4^+^ T cell subsets and immune response mechanism in **Figure 1**.

### CD8^+^ T cells/CTL

Autoreactive cytotoxic CD8^+^ T cells recognize hematopoietic stem and progenitor cell (HSPC) antigens through major histocompatibility MHC I/II, resulting in secretion of pro-inflammatory cytokines such as IFN-γ. After activation, CD8^+^ T cells rapidly proliferate, exit the lymph nodes, enter the bloodstream, and migrate to the infection site. They directly kill target cells by releasing perforin and granzyme or induce apoptosis by using the Fas ligand protein on their surface to bind with the Fas protein on target cells (125–127). Similar to CD4^+^ cells, CD8^+^ cells can be classified into three subtypes based on their distinct phenotypes and functional heterogeneity (128, 129).

### Naïve and memory T cells

T cell subsets serve as indicators of immune function. CD4^+^ cells play a pivotal role in humoral and cellular immune systems by secreting numerous cytokines and facilitating B cell antibody production (130–132). Notably, T cells can be classified as either Tn (CD45RA^+^ and CD45RO^–^) or TM (CD45RA^–^ and CD45RO^+^) based on the expression of these variants (133). Tn cells mature in the thymus and migrate to peripheral secondary lymphatic organs/tissues such as the spleen and lymph nodes (LNs). For Tn, central memory T cell (Tcm), and effector memory (Tem) cells, APC functional differentiation strength increases gradually, while proliferative capacity and antitumor efficacy decline progressively (134). CD8^+^ effector T cell subsets identified thus far include Tc1, Tc2, Tc9, Tc17, follicular cytotoxic T (Tfc), follicular helper T (CD8^+^ Tfh), and regulatory T (CD8^+^ Treg), holds significant potential in treating tumors, viral infections, allergies, and autoimmune diseases (135, 136).The majority of effector cells undergo apoptosis with small subset persists and differentiates into memory cells. Some effector T cells directly transition into Tcm and Tem, while others are converted from Tcm (41, 137, 138). Compared to Tn, TM cells mount and execute a faster immune response upon re-infection by pathogens (137, 139–141).

Memory T cells can be further classified as Tem and Tcm based on the expression of CD62L and CCR7. CD62L, a lymph node homing receptor, influences cell migration, while CCR7, a chemokine receptor, contributes to T cell recirculation and effector function (130, 142, 143). Tcm localize to secondary lymphoid tissues for recirculation similar to Tn cells, whereas Tem preferentially migrate and distribute throughout non-lymphoid tissues and local immune response sites to mount rapid immune responses (144–149). Additionally, Tem cells are involved in early infection stages, while Tcm cells predominate in later stages due to their robust proliferative potential and prolonged effector function (150–159).

Memory cells are vital in vaccine immunity (139). Tcm cells exhibit high expression of CD45RO, CD62L, CD28, CD44, CD11a, and IL-12R (β1 subunit), displaying a stronger proliferative potential and anti-tumor immunity than Tem cells (134, 144, 160) while Tem cells express low levels of CD62L and CCR7. Due to limited CCR7 expression, Tem cells promptly localize in inflamed tissues via chemotactic gradients and express pro-inflammatory factors like IL-4, IL-5, IFNγ, and perforin. Notably, Tem function is implicated in autoimmune diseases and AS development (161–165), evidenced by the Tem cells in synovial fluid or skin during clinical diagnosis (161) and the decrease in Tn cells and increase in Tem cells during AS (166–171), respectively. Tcm cells, akin to stem cells, possess self-renewal capability, while some Tem cells can originate from Tcm in response to antigen stimulation (166–171).

**Figure 2:**
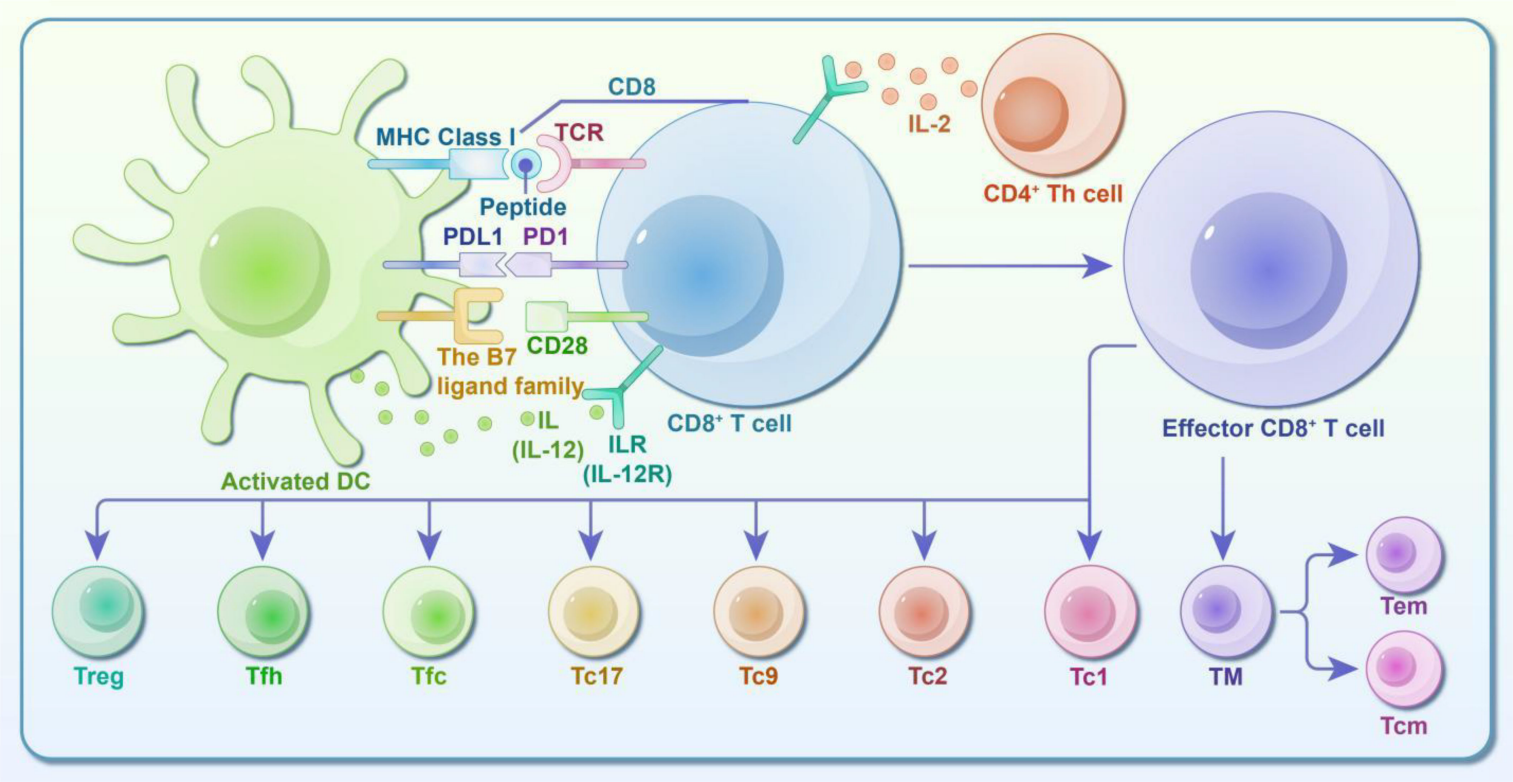
Diagram of CD8^+^ T Cell/CTL subsets and immune response activity. CD8^+^ T cells, also known as cytotoxic T lymphocytes (CTLs), are activated through two primary activities: ① Activation via Antigenic Peptide from APCs: The first signal involves the interaction between the antigenic peptide-MHC I complex and TCR/CD3-CD8 molecules on the APC. The second signal comes from the binding of B7 on the APC to CD28 on the CD8^+^ T cell. Additionally, the T cell expresses the IL-12 receptor, which binds IL-12 secreted by the APC, leading to activation. ② Activation via Antigenic Peptide from Target Cells: In this case, the first signal is provided by the antigenic peptide-MHC I complex binding to TCR/CD3-CD8 molecules. Since target cells lack B7 molecules, CD4^+^ Th cells provide IL-2 to the corresponding receptor on CD8^+^ T cells, serving as the second signal. Upon activation, CD8^+^ T cells gain the ability to differentiate and function. They rapidly proliferate, leave the lymphoid system, enter the bloodstream, and kill target cells. Identified CD8^+^ effector T cell subsets include Tc1, Tc2, Tc9, Tc17, follicular cytotoxic T cells (Tfc), follicular helper T cells (CD8^+^ Tfh), and regulatory T cells (CD8^+^ Treg). These subsets play significant roles in treating tumors, viral infections, allergies, and autoimmune diseases by directly eliminating or inducing apoptosis in target cells. Following the resolution of an infection, most effector cells undergo apoptosis during the contraction phase. However, some CD8^+^ T cells persist as memory cells (TM), which are further categorized into central memory T cells (Tcm) and effector memory T cells (Tem). TM cells are crucial for tumor immunity, host defense, and other immune responses, and they mediate memory responses following vaccination.

In summary, the diagram of CD8^+^ T cell/CTL subsets and immune response mechanism in **Figure 2**.

T cell subsets and their associated functions are summarized in **Table 1**.

**Table 1:** T cell subsets and their associated functions.

T Cell Subsets	Function
CD4^+^T cell	
Th1	secrete pro-inflammatory cytokines like IFN-γ, which mediate cellular immunity and activate other cell types
Th2	secrete L-4, IL-5, and IL-13 thereby supporting the survival and function of B cells
Th9	secrete IL-9, which is closely associated with allergies and autoimmunity
Th17	secrete IL-17, which stimulate neutrophil proliferation and maturation and induce pro-inflammatory cytokines expression in various cell type
Th22	secrete IL-22 which activate several downstream pathways, including MAPK, PI3K/Akt and NF-κB
TFH	assist in the formation of germinal centers and assist in B cell differentiation and maturation
TFR	balance immune activation and tolerance
Treg	maintain peripheral immune tolerance and controlling autoimmune responses
TM	Tem cells exist in lymphoid tissue while Tem cells exist in the peripheral tissues and blood, which react rapidly after antigen stimulation
CD8^+^T cell	
Tc1	secrete IFN-γ and TNF-α directly or indirectlykilling target cells after being stimulated
Tc2	secrete IL-4 IL-5, IL-10 and IL-13, thereby regulating the immune response and mediating allergic reactions
Tc9	secrete IL-9, possess poor cytotaxicity
Tc17	secrete IL-17 and IL-22 possess poor cytotoxicity
TM	Tem cells exist inlymphoid tissue while Tem cells exist in the peripheral tissues and blood, which react rapidly after antigen stimulation

## Platelet regulates T cell immune response

T cells play a pivotal role in both physiological and pathological processes by releasing various cytokines that exert paracrine or autocrine effects on different T cell subsets and other cells, thereby regulating immune responses. Notably, the majority of effector T cells (up to 90 to 95%) undergo apoptosis following antigen clearance (172, 173), posing a challenge for the immune system to maintain long-term self-tolerance during rapid expansions and contractions of cellular population (174). The immune system’s ability to maintain immunological specificity and memory ensures that a small population of memory T cells, which have lower proliferation rates and increased resistance to apoptosis, can persist (175–178). These memory T cells are crucial for initiating rapid and robust immune responses upon re-exposure to the same antigen, forming the backbone of anti-tumor specific immunity.

The mechanisms underlying the persistence of memory T cell remain a topic of debate. While some scientists argue about the necessity of antigen stimulation for memory T cell maintenance (179, 180), the shared surface molecules shared by memory and effector T cells suggest that antigen exposure may indeed play a role in promoting long-term memory. Some reports suggest that weak antigen stimulation can contribute to the persistence of memory T cells (181–183).

Moreover, TM cells are associated with cognate antigen-independent cellular turnover in response to IL-7/IL-15 (154, 184, 185), which supports the sustained maintenance of immune memory (186). An alternative perspective suggests that the longevity of T cells might be directly related to immunological memory itself (186, 187). Additionally, there’s evidence that inflammatory signaling favors the production of effector cells, while the absence of inflammation promotes the development of memory T cells (186).

Platelets are recognized not only for their roles in thrombus formation and hemostasis, but also for their significant regulatory role in immune responses. In collaborating with T cells, platelets release bioactive mediators like PF4 and TGFβ, which contribute to immune modulation and can exacerbate vascular intimal injury (22). Other mediators like PAF, TXA_2_, NAP, and RANTES activate immune cells-such as neutrophils and monocytes/macrophages-promoting chemotaxis and enhancing immune responses through mitochondrial energy metabolism pathways. In concert with CD4^+^ T cells, Platelets influence immune responses by modulating Th and Treg cell phenotypes, affecting the secretion of cytokines like IFN-γ and TNF-α (3, 4, 26, 188).

PF4/CXCL4, TGFβ, and other mediators are secreted by platelet α-granules and are involved in process related to thrombosis, inflammation, and immunity, contributing to vascular intimal injury (22). PF4 exhibits strong chemotactic properties for neutrophils by binding to heparan sulfate on the vascular endothelium, which attenuates thrombin inactivation. TGFβ, predominantly derived from platelets in circulation, serves as a specific marker for *in vivo* platelet activation (2, 23–25).

In the context of inflammatory atherosclerosis, specific macrophage subpopulations have been identified, notably those induced by PF4/CXCL4 and labeled as “M4” (PF4/CXCL4-induced plaque macrophage). The PF4/CXCL4-induced upregulation of MMP7 and S100A8 is mitigated by heparin, which binds to PF4/CXCL4 and glycosaminoglycans, potentially representing macrophage receptors for PF4/CXCL4, characterized by CD68^+^ MMP7^+^ and S100A8^+^ expression (189). studies of atherosclerotic plaque and rheumatoid arthritis synovium have shown that macrophages are a major source of PF4/CXCL4 (190, 191). These interactions between PF4 produced by macrophages and receptors on endothelial cells, fibroblasts, and alveolar type 2 cells suggest significant immune responses, particularly involving PTPRC^+^ immune cells, with PF4 transcripts detected in macrophages based on their expression of CD68, CD163, and MRC1. This indicates that macrophages may be a potential source of PF4/CXCL4 in mouse models of lung and heart fibrosis, as well as in individuals with pulmonary fibrosis (192).

In a study using a pressure overload TAC (transverse aortic constriction) mouse model, PF4 expression was observed in macrophages co-expressing C1q molecules. These macrophages exhibited an M2-like signature, with increased proportions in TAC compared to sham-operated mice after one week (193).

The immune function in chronic inflammatory atherosclerosis involves both innate and adaptive immunity. The innate immunity response is triggered by the accumulation of LDL (low-density lipoprotein) in the arterial wall, which is taken up by macrophages, leading to the formation of microcrystals. This activates inflammasomes, processing of IL-1b, and promoting its secretion, contributing to inflammation and plaque formation. Adaptive immunity plays its part as LDL is transported to arterial draining lymph nodes, where peptide fragments from LDL are presented to T cells, leading to their activation, division, and differentiation. Some T cells stimulate B cells to produce antibodies against LDL, while others contribute to plaque formation and inflammation by activating macrophages, endothelial cells, and smooth muscle cells (194).

Additionally, platelet-thrombocyte aggregates (PTAs) are associated with an increased risk of thrombosis, with cancer patients exhibiting significantly higher percentages of PTAs among CD4^+^ and CD8^+^ T lymphocyte populations compared to healthy individuals (195). Platelets significantly inhibit pro-inflammatory cytokines (IL-12, IL-6, TNFα) while promoting the production of anti-inflammatory cytokine (IL-10) in moDCs (monocyte-derived-dendritic cells) primed with both Toll-like receptor (TLR)-dependent and TLR-independent stimuli. Moreover, platelets and their soluble mediators impede T cell priming and differentiation into the IFNg^+^ Th1 phenotype by moDCs (196).

Interestingly, the absence or inhibition of T cells enhances the antiplatelet effect of clopidogrel by boosting its metabolic activation in the liver, leading to significant production of Cyp2c and Cyp3a in mice. This finding suggests that damage to T cells can enhance the metabolism of drugs that are substrates of Cyp2c or Cyp3a (197).

Platelets possess immunosuppressive properties, releasing anti-inflammatory molecules such as TGF-β and soluble CD40L, which help suppress excessive immune responses and prevent tissue damage. Platelets are the primary source of TGF-β in the human body. This cytokine has been demonstrated to exert deleterious effects on various lymphocytes. Specifically, TGF-β inhibits the differentiation of T cells into cytotoxic T cells while increasing the population of Tregs. Tregs can further inhibit effector T cells and NK cells. TGF-β also directly affects NK cells by impairing their lytic activity and reducing IFN-γ production (198). Therefore, TGF-β released by platelets can inhibit excessive cellular immune responses to prevent tissue damage, its suppression of cellular immunity in tumors can support cancer cell survival.

Platelet-derived CD40 ligand (CD40L) induces production of IL-6 and IL-12 from DCs and enhances their expression of costimulatory molecules such as CD80, CD86 and ICAM-1. Furthermore, CD40L has been shown to enhance DC maturation and their ability to directly kill Staphylococcus aureus, thereby promoting efficient adaptive immunity against the bacterium (199). Besides, platelet CD40L can enhance CD8+ T cell response, hence, functioning as a bridge to the adaptive immune system (200). Additionally, activated platelets can synthesize and secrete IL-1β, a potent pro-inflammatory cytokine. IL-1β up-regulates the expression of adhesion receptors and the secretion of IL-6 and IL-8 in endothelial cells, as well as increases nitric oxide (NO) -induced vascular permeability, thereby playing a significant role in the immune response (201).

SARS-CoV-2 binds to platelet-expressed ACE2/TMPRSS2 through its spike protein, activates the MAPK pathway and enhances platelet activation (aggregation, granule secretion, leukocyte aggregation) and thrombosis, which can be inhibited by recombinant human ACE2 protein and anti-spike monoclonal antibody. The molecular mechanism by which the virus directly drives COVID-19 thromboinflammation is revealed (202). SARS-CoV-2 infection leads to altered platelet gene expression, enhanced activation (manifested as increased P-selectin expression), and increased aggregation with immune cells (neutrophils, monocytes, T cells). Its excessive activation and accelerated aggregation are associated with MAPK pathway activation and increased thromboxane production. In some patients, platelets carry viral mRNA (independent of ACE2), and these abnormalities may exacerbate the pathological process of thrombosis and organ failure in COVID-19 (203).Therefore, platelet immunomodulatory functions reported by studies in SARS-CoV-2 infections, where there has been accumulating evidence that coagulation and complement cascades are deeply interconnected and this can influence immune cell activation.

### Platelets regulate T cell immune response and cytokine release by PF4/TGFβ—via mitochondrial Akt PGC1 α-TFAM signaling pathway

Studies indicate that platelets in healthy individuals selectively enhance CD4^+^ T cell recruitment under normal arterial blood flow conditions. They regulate T effector cell responses through mediators like PF4, TGFβ, and RANTES, which exhibit varying kinetics and effects on the regulation of Th1, Th17, and Treg cells (204).

Mitochondria, the body’s metabolic energy factories, play a crucial role in T cell activation. Within the first 24 to 48 hours after T cell activation, mitochondrial energy metabolism and ATP production are vital for the immune response of T effector cells (205). TFAM (Mitochondrial Transcription Factor A), encoded by nuclear genes, is transported to mitochondria, and serves as a key factor in activating and regulating mitochondrial DNA transcription (3, 4). Additionally, PGC1α (Peroxisome Proliferator-Activated Receptor γ Coactivator 1 α) regulates mitochondrial biosynthesis and proliferation by binding to the downstream target gene NRF-1, which activates its transcription. PGC1α also promotes TFAM expression through co-transcription with NRF-1 and NRF-2, enhancing mitochondrial oxidative function (206).

CXCR3, a functional receptor for PF4, is expressed on CD4^+^ T cells such as Tem and Tcm (3–5, 207). Single-cell RNA sequencing (scRNA-seq) analysis has revealed significant diversity among CD8 T subgroups, particularlly during the peak of influenza virus load and resolution. An enrichment of CXCR3^+^ CD8^+^ T cells correlates with stronger cytotoxic responses. Notably, CXCR3 blockade during late-stage CD8 T cell responses in influenza-cleared lungs can mitigate lung injury without affecting viral clearance, suggesting therapeutic potential for preventing influenza-associated lung injury (208). Moreover, functional CD4^+^ and CD8^+^ T cells exhibiting traits of tissue-resident memory T cells (TRM) have been identified in human kidney tissues, indicating a dynamic immune environment (209). CXCR3 can also enhance specific CD8^+^ T cell activation via plasmacytoid dendritic cells (pDCs) during intracellular pathogen infections (210).

The research highlights that platelets significantly influence T cell subsets (Tem, Tcm, and Tn) and their immune responses, involving mitochondria through PF4 bridging. Key findings include:

1. Platelets significantly impact the Th1/Treg response, with Treg cell response increasing while Th1 responses are inhibited.
2. Platelets undergo PF4-dependent mitochondrial biogenesis and cell proliferation. The Akt-PGC1α-TFAM signaling pathway, initiated by PF4 binding with CXCR3, enhances the responses of Tem and Tcm cells, promoting mitochondrial ATP and ROS production and thus increasing Th1 and Treg responses.
3. Platelets regulate CD4^+^ Tn cell response through PF4-TGFβ interactions. At mildly elevated concentrations, PF4 combined with TGFBRIII promotes TGFβ presentation and TGFBRII expression, enhancing signal transduction and Tn effector cell response. At excessively high concentrations, PF4 can directly bind TGFβ-TGFBRII, blocking TGFβ signal transduction and disrupting the T cell response. This underscores the complex interplay between platelets and T cell regulation, introducing a novel immune regulatory function and highlighting that the platelet-regulated T effector cell response results from multiple factors (3–5, 204).

In conclusion, while platelets are primarily recoginized for their role in blood clotting (hemostasis), they also play significant roles in the immune response. Their involvement is a dynamic process, influenced by various mechanisms, as their interactions with the immune system can modulate responses depending on the context. Thus, the role of platelets in immune activation and suppression is both complex and dynamic.

Platelet activation triggers an increase in oxidative metabolism to meet energy demands, a process that is more efficient than aerobic glycolysis alone. Additionally, platelets exhibit supplemental mitochondrial oxidative phosphorylation, which may serve as a necessary chemical source for platelet activation (211).

However, some studies suggest that:

1. mitochondria derived from platelets can inhibit the proliferation of PBMCs (peripheral blood mononuclear cells).
2. mitochondria from platelets can modulate anti-CD3/CD28-activated CD4^+^ T cells by directly targeting CXCR4 and its ligand SDF-1 (stromal cell-derived factor-1), leading to the upregulation of CD4^+^ Tn and Tcm, while causing a decrease in CD4^+^ Tem (212).

Platelet-based drug delivery strategies have been explored for targeting primary tumors, circulating tumor cells (CTCs), and circulating malignant tumors like lymphoma. Therapeutic agents have been loaded into platelets via endocytosis (e.g., doxorubicin), cell surface chemistry methods (e.g., anti-PD-1 binding), and gene modification (e.g., TRAIL expression). Drug-antibody conjugates have also targeted to target platelet receptors. Given the unique tumor vascular system and the ability of platelets to adhere to circulating tumor cells—especially through surface receptors such as GP IIb/IIIa—innovative targeting strategies have been designed to leverage platelet accumulation in tumors. However, it is still unknown whether this platelet-based drug delivery strategy will cause excessive accumulation of platelets, thereby leading to thrombocythemia and thrombosis formation. More exploration is needed in the future. Alternative approaches include using ligands cleaved by proteins in the tumor microenvironment and surface structures that provide propulsion (213–217).

## Platelet-T cell interaction and impact on AA

AA is a T-cell-mediated bone marrow failure syndrome characterized by the depletion of HSPCs. Research links the activation of T cells to cytokines and chemokines, such as INF-γ, TNF-α, and IL-2, which negatively affect HSPCs, resulting in persistent inhibition of hematopoietic function (218–220). Therefore, IST combined with TPO-RAs (thrombopoietin receptor agonists) or HSCT (hematopoietic stem cell transplantation) is recommended, depending on the patient’s age. Because TPO receptors are expressed on HSPCs, recent research suggests that TPO-RAs can alleviate the inhibitory effects of INF-γ on HSCT in multiple ways, beyond merely raising platelet counts. IFN-γ significantly impacts various T cell subsets, including Th, Treg, and TFH (221, 222). There have been reports of myelofibrosis in patients treated with TPO-RAs. Therefore, patients treated with TPO-RAs should perform bone marrow biopsy once a year/every six months. It is very necessary to be able to discontinue these drugs in a timely manner when grade 2/3 myelofibrosis occurs. Discontinuing TPO-RAs can also prevent the development of clinical manifestations by blocking the progression of grade 2/3 fibrosis (223). Recent study has also shown that Janus kinase (JAK) 1/2 inhibitor ruxolitinib (RUX) can inhibit T cell infiltration activation and inhibit bone marrow cell apoptosis in mice with immune AA. This provides a new idea for the treatment of AA (224).

In children with AA, Treg levels significantly decrease, diminishing their immune suppressive capacity. However, during remission, Treg levels do not show substantial changes (225). New membrane proteins may be identified as platelet sensors for pathogen- or damage-associated molecular patterns (PAMPs and DAMPs), shedding light on novel molecular functions in immunity (226). Furthermore, prior studies have revealed a reduction in platelet-related cytokines in plasma, such as CCL5 and CD40L, reflecting thrombocytopenia in AA. These cytokines are crucial in regulating TH1 and TH2 balance (227).

High levels of PF4 in malignant pleural effusion (MPE) are associated with poor prognosis. The impaired T lymphocyte response caused by PF4 confers an advantage for tumor progression (228).

### Platelets and AA

Platelets can either eliminate microorganisms by direct binding or inhibit their transmission by limiting cell division or survival through indirect effects, which trigger host immune responses. However, many invasive microbial pathogens can target host platelets, directly or indirectly altering platelet counts or function. These microbial pathogens can also influence autoimmunity and alloreactivity in immune-mediated diseases such as immune thrombocytopenia, systemic lupus erythematosus, and multiple sclerosis by interacting with platelet antigens. Conditions like autoimmune thrombocytopenia and fetal and neonatal alloimmune thrombocytopenia are examples of such effects (229).

Platelets have been shown to promote the proliferation of acute leukemia (AL) cells, reduce their sensitivity to chemotherapy, and induce apoptosis. However, the role of platelets in AA remains unclear (230).

Mice lacking CD84, a receptor of the SLAM family, on platelets or T cells, exhibit reduced CD4^+^ T cell infiltration and thrombotic activity in the brain, lowering nerve damage. High platelet CD84 expression is also linked to poorer prognosis in stroke patients. There is overlap in the cytokines and chemokines released by platelets and T cells. Soluble CD48, shed from platelets, can stimulate CD4 T cell migration, and high CD84 expression is associated with an inflammatory immune response (231).

Studies on myeloproliferative neoplasms (MPN) show that PLT interactions with CD8^+^ T cells reduce the proliferation and cytotoxicity of these T cells (232, 233). In malignant pleural effusion, high levels of platelet-derived PF4 are associated with a more severe T lymphocyte response and poor prognosis (227). Mitochondria from platelets can directly interact with CD4^+^ T cells through SDF-1/CXCR4, influencing T cell behavior (212).

A study published in Nature Aging in 2024 revealed that the TRMT6/61A complex, involved in m1A (methylation on the first nitrogen atom of mRNA and tRNA adenosine), drives hematopoietic stem cell aging through a non-methyltransferase pathway. Targeted inhibition of this pathway can delay hematopoietic stem cell aging, which has implications for blood diseases and bone marrow failure. The accumulation of TRMT6/61A in aging HSCs due to deactivated CRL4^DCAF1^ ubiquitin degradation pathway, may disrupt normal blood cell production (234).

Exploring platelet-T cell interactions offers new insights into bone marrow failure treatments. For instance, elevated levels of RANTES and PDPN Mφs have been linked to in severe AA (235). The TPO/Mpl complex regulates megakaryocyte development and platelet production through downstream pathways such as JAK/STAT, Ras/Raf-1/MAPK, and PI3k/Akt. Other regulators, like the interleukin family and IGF-1, can also play supplementary roles in TPO regulation. MicroRNAs, such as miR-9, miR-22, and miR-125a, modulate megakaryocyte and platelet production at various stages. Current treatments like etripopal and romiplimumab aim to boost platelet count by targeting these pathways (236, 237).

### T cells, cytokines, and AA

In a mouse model of AA, an increase in β-chemokines was noted, partly depending on IFN-γ. This cytokine is crucial for upregulating the chemokine receptor CCR5 in macrophages. Blocking CCR5 in murine AA models improved survival, correlating with increased platelet counts and enhanced platelet-biased CD41^hi^ HSCs. While T cells are essential in AA pathogenesis, CCR5 expression on T cells and T cell-derived CCL5 are not necessary for disease progression. In fact, CCR5 antagonism reduces bone marrow macrophages and lower the production of TNF and CCL5, correlating with reduced IFN-γ secretion from bone marrow T cells. Further studies revealed that elderly mice and humans exhibit significantly higher CCR5 expression in macrophages, highlighting CCR5’s role in age-related bone marrow failure. CCR5 signaling plays a crucial role in maintaining bone marrow macrophages, particularly in aging individuals (238).

Additionally, CD8^+^ T cells in AA (aplastic anemia) patients show an activated phenotype characterized by elevated expression of HLA-DR, CD57, and CD27, contributing to hematopoiesis inhibition and bone marrow failure progression. CD38^+^ CD8^+^ T cells, also enriched in both AA patients and animal models, display enhanced pro-inflammatory and proliferative abilities (239–241). Additionally, CD8^+^ GITR^+^ T cells exhibit increased CTLA-4 expression, resulting in a reduced cytotoxic phenotype (242).

CD4^+^ Th1 cells play a significant role in the pathogenesis of bone marrow failure by secreting pro-inflammatory cytokines like IFN-γ and TNF-α, which mediate HSPC apoptosis (216). IFN-γ, in particular, is critical in cellular immunity, as it inhibits precursor cell proliferation *in vitro*, and induces Fas expression on HSPCs. Once Fas is expressed, activated T cells trigger apoptosis through the Fas/FasL pathway, which ultimatly leads to bone marrow failure. In transgenic mice with elevated levels of IFN-γ, early signs of bone marrow aging manifest both in the bone marrow and peripheral blood. These mice exhibit the symptoms characteristic of bone marrow dysfunction and immune dysregulation (243, 244).

Interestingly, despite the significant elevation of IL-18 (a cytokine that is also induced by IFN-γ) in severe AA patients, studies have shown that IL-18 gene knockout in mouse models does not prevent bone marrow failure. This suggests that while IL-18 may be involved in the inflammatory response, it does not directly drive the pathogenesis of AA (243).

Additionally, TNF-α is a key negative regulator of hematopoiesis. It acts through its receptors on T cells and bone marrow CD34^+^ cells, further contributing to cell damage and the progression of bone marrow failure (245–247).

Thes studies underscore the complex interplay of cytokines like IFN-γ, TNF-α, and IL-18 in the immune-mediated destruction of hematopoietic cells, with IFN-γ playing a central role in inducing bone marrow failure through the Fas/FasL pathway and inhibiting progenitor cell proliferation.

### T cell dysfunction is closely related to the physiological and pathological status of bone marrow failure, and there are also many associations between CD4^+^ T cells and CD8^+^ T cells

CD4^+^ Treg cells in the tumor microenvironment (TME) express high levels of PD-1 (programmed cell death protein 1), suggesting that PD-1 blockade might enhance Treg’s immunosuppressive function. Research has indicated that anti-PD-1 monoclonal antibodies (mAb), commonly used in immune checkpoint inhibition therapy, may paradoxically boost Treg-mediated immunosuppressive activity in cancer patients. Moreover, Treg cells deficient in PD-1 deficiency exhibit an even more potent ability to suppress immune responses (248, 249). Conversely, PD-1 expression is also a hallmark of exhausted CD8^+^ T cells, which limits their cytotoxic function due to chronic TCR (T cell receptor) stimulation. Immune checkpoint inhibitors (ICIs), like anti-PD-1 or anti-PD-L1 mAb, block the interaction between “PD-1—PD-L1 (PD-1 ligand)”, effectively restoring cytotoxic capabilities of CD8^+^ T cells. This restoration helps in reducing viral load and tumor progression, as demonstrated in clinical practice across various cancer treatments (250–255).

In addition, CXCR5+ CD8+ T cells have emerged as crucial players in immune regulation, particularly within the TME and during chronic infections. These cells exhibit dual functions: they not only assist B cells in germinal centers in a manner similar to CXCR5+ CD4+ T follicular helper (Tfh) cells by promoting antibody production, but they also retain cytotoxic activity, crucial in infection and cancer contexts (254–258). CXCR5+ CD8+ T cells, like their CD4+ counterparts, also express high levels of PD-1, and may exhibit an “exhausted” phenotype in the tumor microenvironment, making them potential targets for ICIs (256–260).

**Figure 3:**
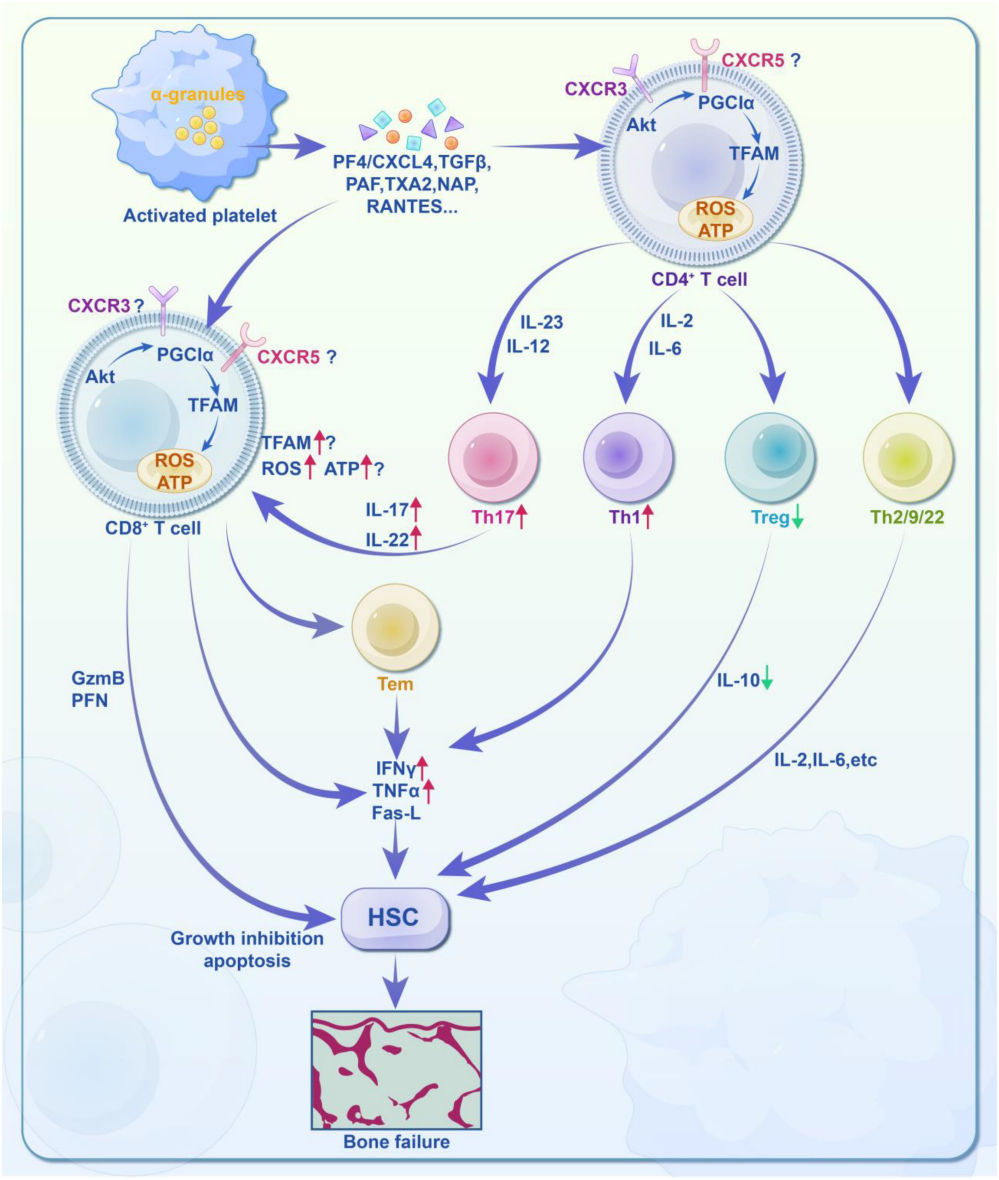
Molecular activity of platelet-regulated T cell immune response in acquired aplastic anemia (AA). AA may be due to an unknown pathogen infecting hematopoietic stem cells (HSCs) or peripheral cells, leading to the presentation of pathogen particles and either unmodified or chemically/genetically modified components on their cell surfaces. These antigens are then presented to antigen-presenting cells (APCs), which process and present them to CD4^+^ T cells. Platelets play a role in this immune regulation by releasing various soluble mediators, such as PF4/CXCL4, TGFβ, PAF, TXA2, NAP, and RANTES. PF4 binds to CD4^+^ or CD8^+^ T cell/CTL membrane receptors CXCR3 and CXCR5, activating the downstream mitochondrial energy metabolism signaling pathway (Akt-PGC1α-TFAM). This affects mitochondrial quantity, ATP production, and reactive oxygen species (ROS), thereby regulating the T cell immune response. For CD4^+^ T cells: ① Differentiation into Th1, Th2, Th9, and Th22 phenotypes. ② Simultaneous differentiation into Th17 phenotypes under the stimulation of IL-23 and IL-12. ③ Suppression of Treg phenotypes, leading to weakened immune regulation and immune imbalance. For CD8^+^ T cells: ① Direct cytotoxicity through the release of granzyme B (GzmB) and perforin (PFN). ② Paracrine effects via TNFα, IFNγ, and Fas ligand (Fas-L). In summary, platelets regulate the immune responses of CD4^+^ and CD8^+^ T cells through mitochondrial energy metabolism, contributing to immune imbalance and increased levels of IFNγ and TNFα. This results in the attack on HSPCs and may ultimately lead to bone marrow failure.

In the context of thromboinflammation, activated platelets expressing integrin α_IIb_β_3_ and P-selectin are known to contribute to platelet aggregation, endothelial damage, and microthrombosis. This interaction increases the formation of neutrophil extracellular traps (NETs), which amplify cytokine release and further inflammation. Given this, future therapeutic strategies targeting thromboinflmmatory responses are focusing on dieases such as thrombosis, sepsis, influenza, and COVID-19 (261). Advances in platelet-T cell immune regulation may also enable novel drug delivery systems, such as the use of platelet-coated gold nanoparticles to modulate the tumor microenvironment (217, 262) (**Figure 3**).

## Summary and future perspective

**Figure 4:**
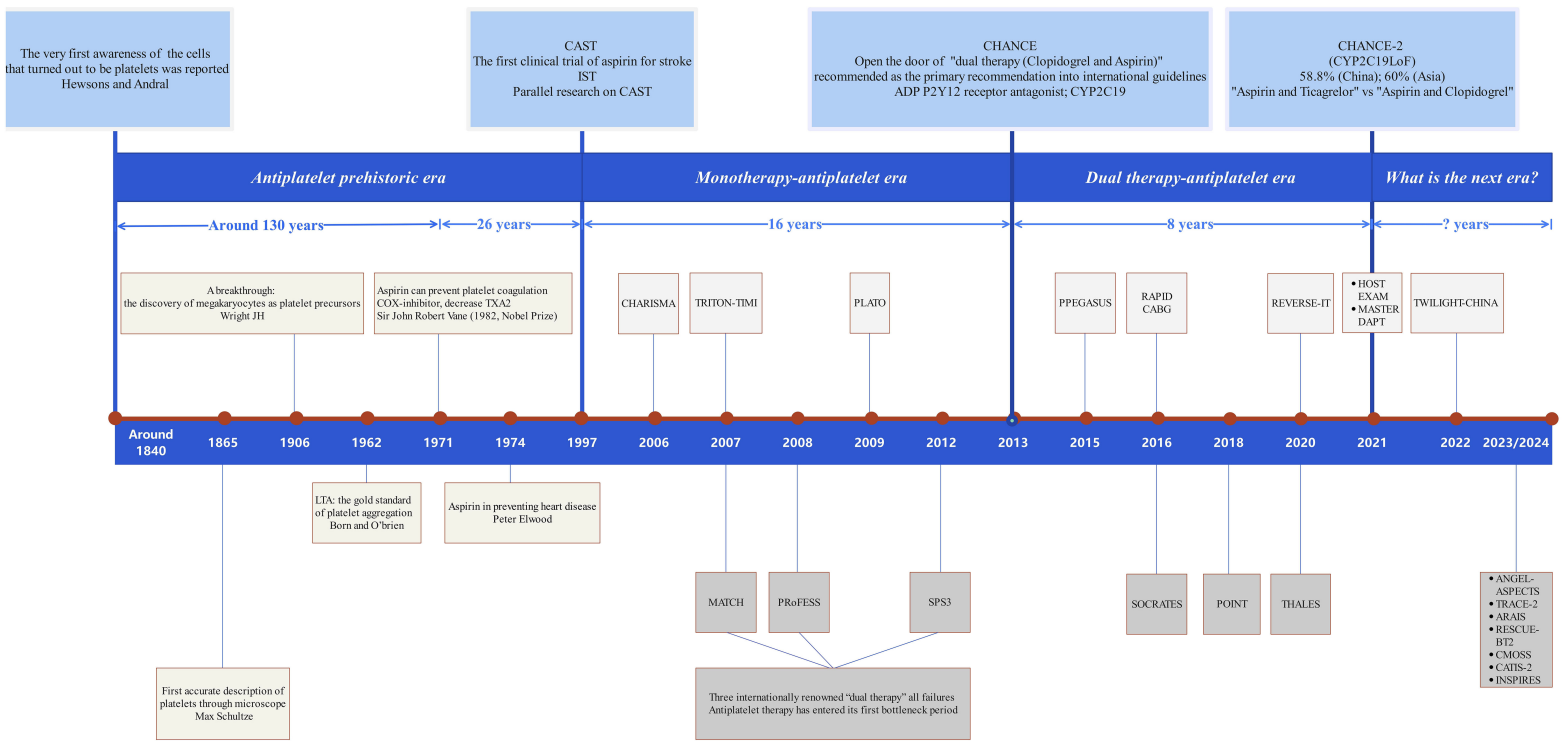
Diagram of the brief history of platelet drugs. The brief history of platelet drugs and antiplatelet therapy spans over 180 years and can be divided into four key stages: ① Initial Development Stage (The Concept of Platelets): This stage marks the initial understanding of platelets and their role in clotting. ② Mono-Antiplatelet Stage (Introduction of Aspirin): The development of aspirin as the first antiplatelet drug represents a significant milestone in antiplatelet therapy. ③ Dual-Antiplatelet Stage (Combination Therapy): This stage involves the combination of two antiplatelet drugs to enhance therapeutic efficacy. ④ Current New Era Stage (Immune Targeting and Precision Medicine): Advances in personalized medicine and immune targeting characterize this latest phase. The CHANCE2 study highlights that suitable antiplatelet drugs, such as ticagrelor or clopidogrel, can be selected by detecting CYP2C19 loss-of-function mutations. Despite ticagrelor providing only 2% greater protection for primary endpoints compared to clopidogrel within one year, it appears we have reached a new bottleneck in antiplatelet therapy. Thus, identifying new targets for platelet drugs remains a crucial goal for future research.

The development of platelet drugs and antiplatelet therapies has progressed through four key stages: the prehistoric phase, focusing on the conceptualization of platelets; the mono-antiplatelet phase, marked by the introduction of aspirin; the dual-antiplatelet phase, involving the combination of two antiplatelet drugs; and the current new era, characterized by immune targeting and precision medicine (**Figure 4**). The CHANCE2 study has enabled the selection of appropriate antiplatelet drugs, such as ticagrelor or clopidogrel, based on detecting CYP2C19 gene loss-of-function mutations (263).

Despite advancements, the efficacy of antiplatelet drugs has hit a plateau. Improvements, even with widely used drugs like aspirin, clopidogrel, and ticagrelor, have only reached about 2%. This highlights the need for new targets and directions beyond traditional COX inhibitors or ADP P2Y12 receptor antagonists.

Interestingly, recent studies suggest that platelet function extends beyond their roles in thrombosis and hemostasis, playing significant roles in immune regulation. As the second most abundant blood cell type, platelets engage with T cells through mitochondrial metabolic pathways, influencing T cell development, proliferation, differentiation, survival, and apoptosis.

Platelets activate the downstream mitochondrial energy metabolism signaling pathway (Akt-PGC1α-TFAM) of T cells by releasing various soluble mediators such as PF4/CXCL4, TGFβ, PAF, TXA2, NAP and RANTES, affecting the number of mitochondria, ATP production and reactive oxygen species (ROS), thereby regulating the immune response of T cells. CD4 + T cells differentiate into Th1, Th2, Th9, Th17 and Th22 phenotypes, while the Treg phenotype is inhibited, resulting in weakened immune regulation and immune imbalance. CD8 + T cells, on the one hand, directly produce cytotoxicity by releasing granzyme B (GzmB) and perforin (PFN), and on the other hand, exert paracrine effects through TNFα, IFNγ and Fas ligand (FAS-L). These immune responses promote immune imbalance and elevated levels of IFNγ and TNFα. This leads to an attack on hematopoietic stem cells and may eventually result in bone marrow failure.

In AA, platelets may regulate T cells through several mechanisms: 1) Mitochondria-mediated regulation of T cells via the Akt-PGC1α-TFAM signaling pathway leads to increased mitochondrial biogenesis, boosting ATP and ROS production. This may result in immune imbalance, overactivation of CD4^+^ Th1, suppression of Tregs, and imbalance in CD8^+^ T cell phenotypes (e.g., CD38, PD-1, GITR, CTLA-4, HLA-DR, CD57, CD27), contributing to elevated IFN-γ and TNF-α levels and subsequent HSPC damage. 2) Platelet-derived mediators like PF4 and TGFβ can modulate PF4-TGFβ dual signaling. Low PF4 levels increase TGFβ signaling through TGFBRIII, while high levels inhibit this pathway, potentially triggering excessive immune response and damage to hematopoietic stem cells.

Further research is required to understand how telomere dysfunction, hematopoietic microenvironment abnormalities, and other immune cells contribute to AA. Investigating these mechanisms may guide future clinical treatments.

Overall, the emerging understanding of platelet-T cell interactions, combined with platelet-based drug delivery technologies, could lay a strong foundation for immune-inflammatory therapies. Platelets may become a central focus in research on atherosclerosis and immune-related inflammation, garnering significant interest from the scientific community (**Figure 4**).
